# Regulatory Basis of Adipokines Leptin and Adiponectin in Epilepsy: from Signaling Pathways to Glucose Metabolism

**DOI:** 10.1007/s11064-023-03891-2

**Published:** 2023-02-16

**Authors:** Yisi Shan, Yeting Chen, Haiping Gu, Yadong Wang, Yaming Sun

**Affiliations:** 1 Department of Neurology, Zhangjiagang TCM Hospital Affiliated to Nanjing University of Chinese Medicine, Zhangjiagang, 215600 China; 2 Translational Medical Innovation Center, Zhangjiagang TCM Hospital Affiliated to Nanjing University of Chinese Medicine, Zhangjiagang, 215600 China; 3 Department of Acupuncture, Zhangjiagang Second People’s Hospital, Zhangjiagang, 215600 China

**Keywords:** Adipokines, Leptin, Adiponectin, Epilepsy, Signaling pathways

## Abstract

Epilepsy is a common and severe neurological disorder in which impaired glucose metabolism leads to changes in neuronal excitability that slow or promote the development of epilepsy. Leptin and adiponectin are important mediators regulating glucose metabolism in the peripheral and central nervous systems. Many studies have reported a strong association between epilepsy and these two adipokines involved in multiple signaling cascades and glucose metabolism. Due to the complex regulatory mechanisms between them and various signal activation networks, their role in epilepsy involves many aspects, including the release of inflammatory mediators, oxidative damage, and neuronal apoptosis. This paper aims to summarize the signaling pathways involved in leptin and adiponectin and the regulation of glucose metabolism from the perspective of the pathogenesis of epilepsy. In particular, we discuss the dual effects of leptin in epilepsy and the relationship between antiepileptic drugs and changes in the levels of these two adipokines. Clinical practitioners may need to consider these factors in evaluating clinical drugs. Through this review, we can better understand the specific involvement of leptin and adiponectin in the pathogenesis of epilepsy, provide ideas for further exploration, and bring about practical significance for the treatment of epilepsy, especially for the development of personalized treatment according to individual metabolic characteristics.

## Introduction

Adipokines, containing various bioactive peptides/proteins, immune molecules and inflammatory mediators, are secreted by adipose tissue and normally function through autocrine, paracrine and endocrine signaling [1]. Leptin and adiponectin, as two pivotal adipokines, act extensively in the central nervous system, and play an important role in the pathophysiology of different neurological diseases, including epilepsy [2–6].

Leptin, as a vital regulator in different physiopathologic processes, binds to leptin receptors and activates signaling pathways in the regulating different cellular functions to be neuroprotective. Although mice deficient in the mitochondrial manganese superoxide dismutase (MnSOD or SOD2) exhibit spontaneous seizures [7], leptin induces the production of MnSOD and the anti-apoptotic protein B-cell lymphoma-extra large (Bcl-XL), stabilizes the mitochondrial membrane potential and alleviates mitochondrial oxidative stress [8]. Mice deficient in leptin receptors are more prone to hippocampal damage caused by epilepsy, and intraventricular administration of leptin protects hippocampal neurons [8]. Electrophysiological and biochemical tests have shown that leptin has an anticonvulsant effect on pentylenetetrazol (PTZ) -induced generalized tonic–clonic convulsive seizure in the Wistar rat model. Leptin significantly increases the serum endogenous anticonvulsant agent galanin and glutathione (GSH). Besides, it decreases the expression level of malondialdehyde (MDA), which may be protective against oxidative damage [9].

Adiponectin is the 30 kDa adipocyte complement-related protein (Acrp30). Its receptors cover many biological functions. Adiponectin receptor 1 (AdipoR1) and receptor 2 (AdipoR2) have physiological correlations in metabolic processes. T-cadherin, a cadherin superfamily member, is a potent receptor for hexamer and adiponectin oligomers with high molecular weight [10–12]. Full-length adiponectin is cleaved by leukocyte esterase to form globular adiponectin (gAd) [13]. AdipoR1 has a high affinity for gAd, compared with AdipoR2 for full-length and gAd as an intermediate-affinity receptor [14]. Adiponectin transcription is regulated by Sirtuin 1/forkhead box protein O 1 (FoxO1) and peroxisome proliferator-activated receptors (PPARs) [15]. Adiponectin exerts a neuroprotective effect on brain damage in different regions through AdipoR1 [16–20] and, especially in the hippocampus, promotes neurogenesis through AdipoR1 [21] and directly affects synaptic function by AdipoR2 [22]. Adiponectin deficiency in mice on a high-fat diet results in increased seizure severity and pathological changes in the hippocampus [19].

Via bioinformatics technology, researchers used Gene Ontology (GO) and the Kyoto Encyclopedia of Genes and Genomes (KEGG) network to reveal that synapses play a crucial role in medial temporal lobe epilepsy (MTLE) [23]. It has been reported that the changes of postsynaptic glutamate receptor increase the excitability of hippocampal neural network, and 4-aminopyridine (4-AP) -induced epileptiform activity in hippocampal brain slices of rat in vitro model shows that alpha-amino-3-hydroxy-5-methyl-4-isoxazolepropionate (AMPA)/N-methyl-D-aspartate (NMDA) ratio increases [24]. Using a cell model cultured with low magnesium, it has been observed that prolonged epileptiform activity increases reactive oxygen species (ROS) production in an NMDA receptor-dependent manner, further resulting in neuronal damage and apoptosis induced by epilepsy [25]. It has been described that oxidative damage in surgically resected brain tissue with epilepsy [26, 27]; conversely, oxidative damage may affect neuronal excitability and susceptibility to suffer from epilepsy [28–30].

Seizures and their potential effects on the development of the brain, especially for patients with clinically intractable epilepsy, may experience varying degrees of cognitive impairment, behavioral abnormalities, or psychiatric symptoms, all of which cause severe limitations in daily lives [31, 32]. However, many scholars have devoted to study the regulatory mechanism of leptin and adiponectin which has provided unique insights and opened a new perspective for the pathogenesis and treatment of epilepsy. This article mainly discusses the role of leptin and adiponectin in the genesis of epilepsy and their effects on antiepileptic drug treatment, in order to find an intervention to regulate signal transduction to control the progression of epilepsy and improve the quality of life in patients with epilepsy.

## Epilepsy

Dysfunction in metabolic processes can bring about changes in neuronal excitability [33], promoting or alleviating seizure progression. Proteomic technology to screen for differential molecules associated with seizures has indicated that most of the affected proteins involve in energy metabolism and redox balance [34]. This article will discuss the correlation among various biological functions and complex signaling mechanisms of leptin and adiponectin, the way how these two adipokines regulate the metabolism and energy homeostasis and the pathological processes of epilepsy.

Febrile seizures(FS) is a common convulsive disease in children. Low-level leptin in cerebrospinal fluid (CSF) is related to the susceptibility to complex febrile seizures [35]. Chronic deficiency of leptin increases the susceptibility to seizures, severity, and possibility of suffering from generalized clonic and clonic-tonic seizures in PTZ-induced models [36]. However, current evidence has suggested that adiponectin is specifically expressed in different types of epilepsy. The logistic regression analysis has shown that a high serum adiponectin level is a significant risk factor for FS [37]. Inconsistently, serum adiponectin level reduces in adults with temporal lobe epilepsy (TLE) and in patients with refractory epilepsy [4, 38]. A study for 13 female patients has indicated that the plasma adiponectin levels are significantly increased within 24 h after primary or secondary generalized tonic–clonic seizures [39].

The pathogenesis of epilepsy starts from pathophysiological changes to the progression after seizures, including changes in voltage and transmitter-gated channels, intracellular signal cascades, synaptic connection, alteration in gene expression, abnormal protein production and activation or inhibition of the metabolic pathway, etc., which may be targeted indicators for the drug to inhibit epileptogenesis.

## The Effects of Leptin on Epilepsy

### The Signaling Cascade of Leptin

**Fig. 1:**
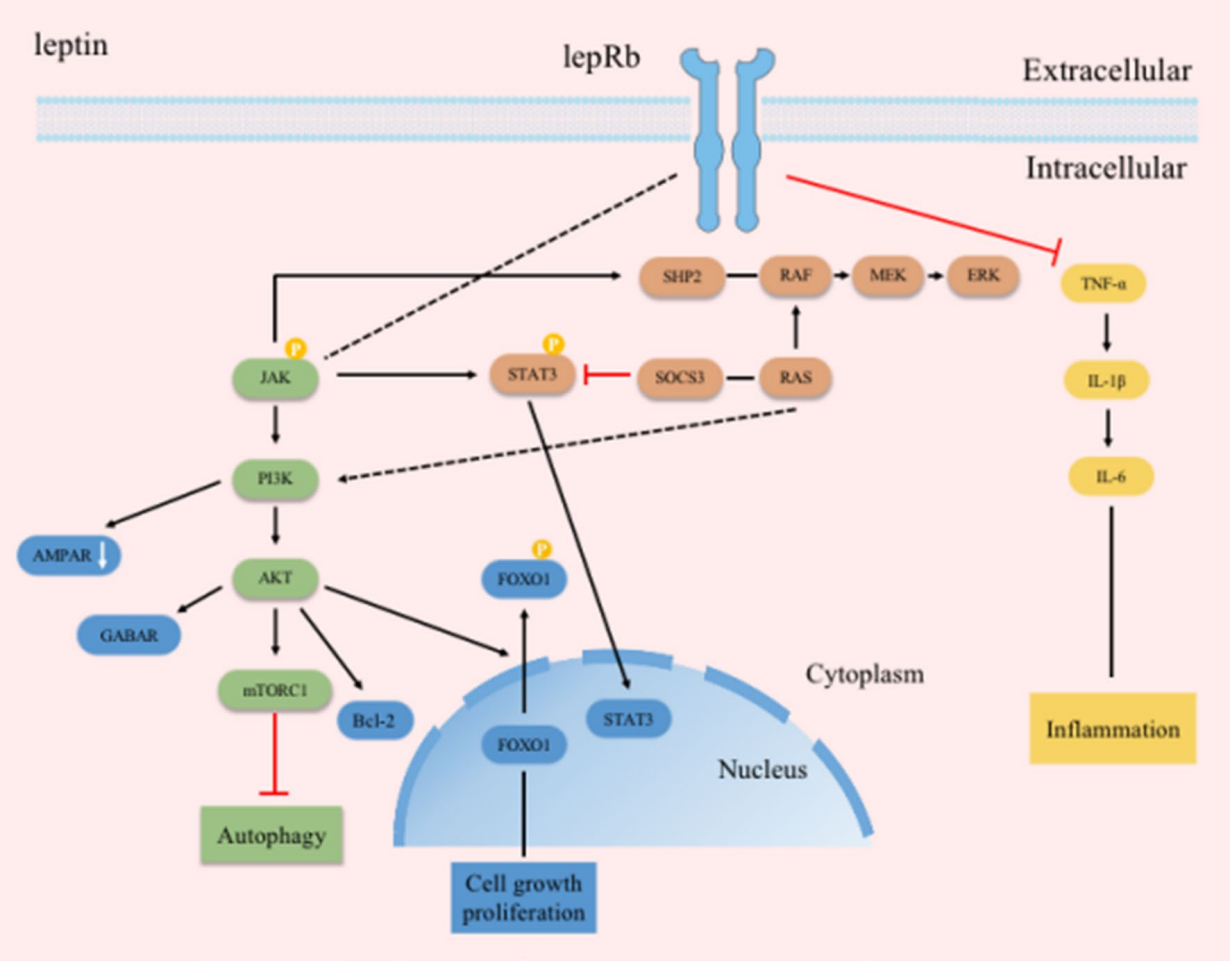
Leptin receptors participate in multiple signaling pathways

Leptin is a peptide hormone derived from adipocytes. Leptin receptors are expressed in both neonatal and adult hippocampal neurons, and through the blood–brain barrier (BBB) act in many regions of the central nervous system, where they involve in the regulation of energy balance, inflammatory processes, synapse formation and neurotrophic activity [9, 40–43]. Leptin binds to long-form receptors at the plasma membrane, to trigger multiple signaling cascades (Fig. 1), including Janus kinase-signal transducer and activator of transcription (JAK-STAT) signaling, phosphatidylinositol 3-kinase (PI3K)/protein kinase B(Akt)/FOXO1 signaling, and Src homology 2 domain-containing protein tyrosine phosphatase 2 (SHP2)- extracellular signal-regulated kinase (ERK) signaling.

The JAK-STAT pathway is a major signaling mechanism for various cytokines and growth factors. As a family of non-receptor tyrosine kinases, JAK activates and stimulates cell proliferation and apoptosis. After being phosphorylated by JAK, the substrate STAT dimerizes and crosses the nuclear envelope into the nucleus to regulate the expression of related genes [44]. In addition, receptors phosphorylated by JAKs recruit PI3K to activate the PI3K-Akt pathway. Akt phosphorylates target proteins through various downstream pathway to play a role in inhibiting apoptosis [45–47]. For example, Akt phosphorylates FoxO1, a transcription factor of the FoxO family, at multiple sites, resulting in the translocation of FoxO1 from the nucleus to the cytoplasm.

### The Regulatory Mechanisms of Leptin in Glucose Metabolism

Glucose metabolism disorder affects the progression of epilepsy [48, 49]. Leptin, as an essential mediator of metabolic homeostasis, regulates glucose homeostasis both externally and centrally, and the peripheral targets are pancreatic β-cells. Depending on NMDA receptors, calcium/calmodulin-dependent kinase β (CaMKKβ) and AMP-activated protein kinase (AMPK), leptin increases cell membrane protein kinase A (PKA) activity, induces ATP-sensitive potassium (K_ATP_) channels transport to the β-cells surface to inhibit glucose-stimulated insulin secretion, thereby increasing K^+^ conductance and causing β-cells hyperpolarization [50].

Studies have demonstrated increased glucose uptake in the brain after intravenous leptin administration in wild-type mice [51]. Leptin in neurons influences cellular glucose uptake through glucose transporters [52, 53]. Leptin also enhances lactate dehydrogenase A (LDHA) -dependent glucose perception in the hypothalamus to regulate glucose production in high-fat-fed rodents [54]. However, for 18 h food-deprived rats, microinjection of glucose in the hypothalamic paraventricular nucleus (PVN) can reduce plasma leptin levels [55]. The PI3K signaling pathway in hypothalamic neurons integrates leptin and insulin to coordinate systemic glucose and energy homeostasis [56]. Research has found that agouti-related peptide (AGRP) neurons play a role in the leptin regulation of energy balance and glucose homeostasis [40]. Potential mechanisms for its neurobiological effects include presynaptic enhancement of gamma-aminobutyric acid (GABA) neurotransmission and postsynaptic activation of adenosine triphosphate (ATP) -sensitive potassium channels. Continuous activation of leptin receptor neurons in the arcuate nucleus of the hypothalamus leads to impaired glucose tolerance [57]. Leptin receptors in the commissural nucleus of the tractus solitarius (cNTS) induce brain glucose retention (BGR) by enhancing hypoxic stimulation of carotid chemoreceptors [58].

### Dual Roles of Leptin in Epilepsy

Leptin involves in energy metabolism through the PI3K-Akt-FoxO1 pathway in neurons [59]. However, the antiepileptic drug valproic acid (VPA) promotes the phosphorylation of Akt and FoxO1 [60]. Studies have shown that leptin activates the JAK/STAT and PI3K-Akt pathway and promotes neuronal survival by increasing the production of the antioxidant enzyme Mn-SOD and the anti-apoptotic protein Bcl-XL [8]. PI3K/Akt/Mechanistic target of rapamycin (mTOR) pathway may cause abnormal transduction of neuronal signal in epilepsy under pathological conditions [61–63].

The expression of Jak1, Stat1 and Stat3 in the hippocampal tissue of epileptic rats induced by lithium-pilocarpine increases [64]. Status epilepticus (SE) activates the JAK/STAT pathway [65], and selective inhibitors of the JAK/STAT pathway administered within 1 h after the onset of SE lead to transient suppression of STAT3 phosphorylation (pSTAT3) and a long-term reduction in the frequency of spontaneous seizures. In the study based on chromatin immunoprecipitation and chromatin immunoprecipitation followed by sequencing (ChIP-seq) [66], Lesiak et al. found that the suppressor of cytokine signaling 3 (SOCS3) is a direct target of the Cyclic AMP response-element binding protein (CREB) transcription factor.Leptin activates the Mitogen-activated protein kinase (MAPK) kinase (MEK)/ERK pathway and upregulates SOCS3 expression through the CREB transcription factor, thereby increasing synaptogenesis in hippocampal neurons [43]. The mechanism of leptin and STAT3 may depend on the interaction between SHP2 and SOCS3. After leptin receptors are activated, the conserved tyrosine residues on its tail are phosphorylated by JAK2 to promote the aggregation of downstream signaling proteins. Cytoplasmic tyrosine residue Tyr^985^ phosphorylates to combine with SHP2 which contains Src homology2 (SH2) domains and SOCS3. SHP2 binds phosphorylated Tyr^985^ and mediates the activation of ERK in cultured cells. SOCS3 mediates feedback inhibition of LepRb signaling by binding to Tyr^985^. SHP2 acts as a competitive and negative regulator in the processes of SOCS3's binding to Tyr^985^ associated with leptin receptors [67, 68]. SHP2 contains phosphatase domains and tyrosine phosphorylation sites. It plays a part in the regulation of cellular transduction pathway related to cytokine, growth factors and hormones, especially rat sarcoma (RAS)/MAPK and PI3K/AKT cascades. SHP2 dephosphorylates RAS and enhances its binding to the effector protein rapidly accelerated fibrosarcoma (RAF), thereby activating the downstream MEK/ERK signaling pathway [69]. MEK1 expression in the mouse brain not only leads to ERK activation to bring about spontaneous seizures, but also to phosphorylation of the transcription factor CREB [70]. Nguyen LH et al. [71] found that MEK inhibitor PD0325901 (mirdametinib) significantly decrease seizure activity in tuberous sclerosis complex (TSC) mouse models.

In addition to the abnormal release of neurotransmitters, epilepsy is closely related to the highly synchronized abnormal firing of neurons caused by abnormally transmembrane movement of ions. The changes in the structure and function of ion channels lead to excitatory regulation disorder to induce epilepsy. Leptin, however, plays an essential role in different epileptic models. Researchers have found that leptin counteracts the up-regulation of the protein level of the Zn (2 +)/Ca(2 +) signaling, which has a neuroprotective effect in the pilocarpine-induced neonatal Sprague–Dawley rat status epilepticus model [72] and inhibits the excitability of hippocampal neurons by activating Ca^2+^- and voltage-gated K^+^ channels of large conductance (BK channels) through PI3K [73]. The duration and incidence of focal seizures induced by 4-AP, an inhibitor of voltage-gated K^+^ channels, decrease after neocortical injection of leptin. Intranasal administration of leptin in mice delays the seizures of generalized convulsions induced by the chemical convulsant PTZ [74]. Leptin not only plays a potentially neuroprotective role by reducing cell damage related to SE induced by kainic acid (KA) [75], but also lowers the neuronal spiking in an in vitro epilepsy model and inhibits alpha-amino-3-hydroxy-5-methyl-4-isoxazole propionic acid (AMPA) receptor-mediated synaptic transmission in the mouse hippocampus [74]. Ligand-gated ion channels, glutamate receptors mediate excitatory synaptic transmission in the central nervous system, and leptin directly affects glutamate neurotransmission in the hippocampus to inhibit seizures [74]. In hippocampal astrocytes of epileptic mice [76], pretreatment with leptin reduces the toxicity of excess glutamate to glial cells and plays a protective role against seizures. Leptin treatment improves the neurobehavioral abnormality generated by Flurothyl-induced recurrent seizures. Moreover, long-term treatment with leptin reverses the up-regulation of Beclin-1/Bcl-2 protein level and the down-regulation of Ca^2+^/calmodulin-dependent protein kinase IIα (CaMKIIα) level [77]. Other evidence has shown that leptin reduces the expression of proinflammatory cytokines tumor necrosis factor-α (TNF-α), interleukin-1β (IL-1β) and interleukin 6 (IL-6) levels, suggesting that leptin may have an anti-inflammatory effect upon epileptic seizures [9].

Notably, studies have reported the proconvulsant activity of leptin. Intraventricular administration of leptin at a dose of 1 μg in a rat model with penicillin-induced epilepsy increases the mean frequency of epileptiform activity, but never changes its amplitude [78]. Electrophysiological studies have proven that inhibition of cannabinoids can mediate leptin's convulsant-stimulating activity [79]. Activating the cannabinoid receptors type 1 (CB1) mediates the anticonvulsant effect of cannabinoids [80]. Intraventricular injection of 7.5 μg CB1 agonist arachidonyl‐2‐chloroethylamide (ACEA) protects against penicillin-induced epileptoid activity. However, leptin blocks this effect and enhances the convulsant-promoting effect of [N‐(piperidine‐1‐yl)‐5‐(4‐iodophenyl)‐1‐(2,4‐dichlorophenyl)‐4‐methyl‐1H‐pyrazole‐3 carboxamide] (AM‐251), a cannabinoid CB1 receptor antagonist [79]. Moreover, leptin blocks glucocorticoid-mediated endocannabinoid release in the paraventricular nucleus of the hypothalamus through phosphodiesterase 3B-mediated reduction of intracellular cyclic adenosine monophosphate (cAMP) levels [81]. In addition, such changes may be related to the involvement of the neuronal nitric oxide synthase (NOS)/nitric oxide (NO) pathway in mediating the seizure-like activity in the processes mentioned above. nNOS increases γ-aminobutyric acid transaminase (GABA-T) activity and reduces brain GABA levels, and the NO produced may activate NMDA receptors [82]. Leptin increases NMDA (dual voltage and transmitter-gated channel) receptor-mediated synaptic currents and triggers N-methyl-D-aspartate receptor (NMDAR) -dependent Ca^2+^ influx [83]. Experimental research [84] has described that leptin exhibits dose-related proconvulsant activity with NMDA and KA, including decreasing latency and increasing symptoms.

Although leptin has shown inhibitory and neuroprotective activity against seizures in several epileptic models, it also increases epileptiform activity in other models under certain conditions. Reports on leptin's pro-convulsant and anticonvulsant effects have indicated specific roles of leptin in different epilepsy models and signaling pathways.

At present, antiepileptic drugs are still the mainly clinical treatment for epilepsy [85, 86]. Among children receiving long-term treatment with VPA, carbamazepine (CBZ) and lamotrigine (LTG), the serum leptin level remarkably increases in the VPA group [87]. Administration of antiepileptic drugs for at least 6 months for the over-6 age group with idiopathic epilepsy has shown VPA-treated children have higher leptin concentration and a lower ratio of soluble leptin receptor (SOB-R) to leptin [88]. Among children with idiopathic epilepsy or location-related idiopathic epilepsy in the monotherapy with VPA or topiramate (TPM) for at least 6 months [89], the leptin levels in the VPA group are higher than that in the TPM group. Some researchers believe that changes in leptin expression levels may also be one of the anticonvulsant mechanism of ketogenic diet (KD) [90, 91].

## The Effects of Adiponectin on Epilepsy

### The Involvement of Adiponectin in Signaling Cascade

**Fig. 2:**
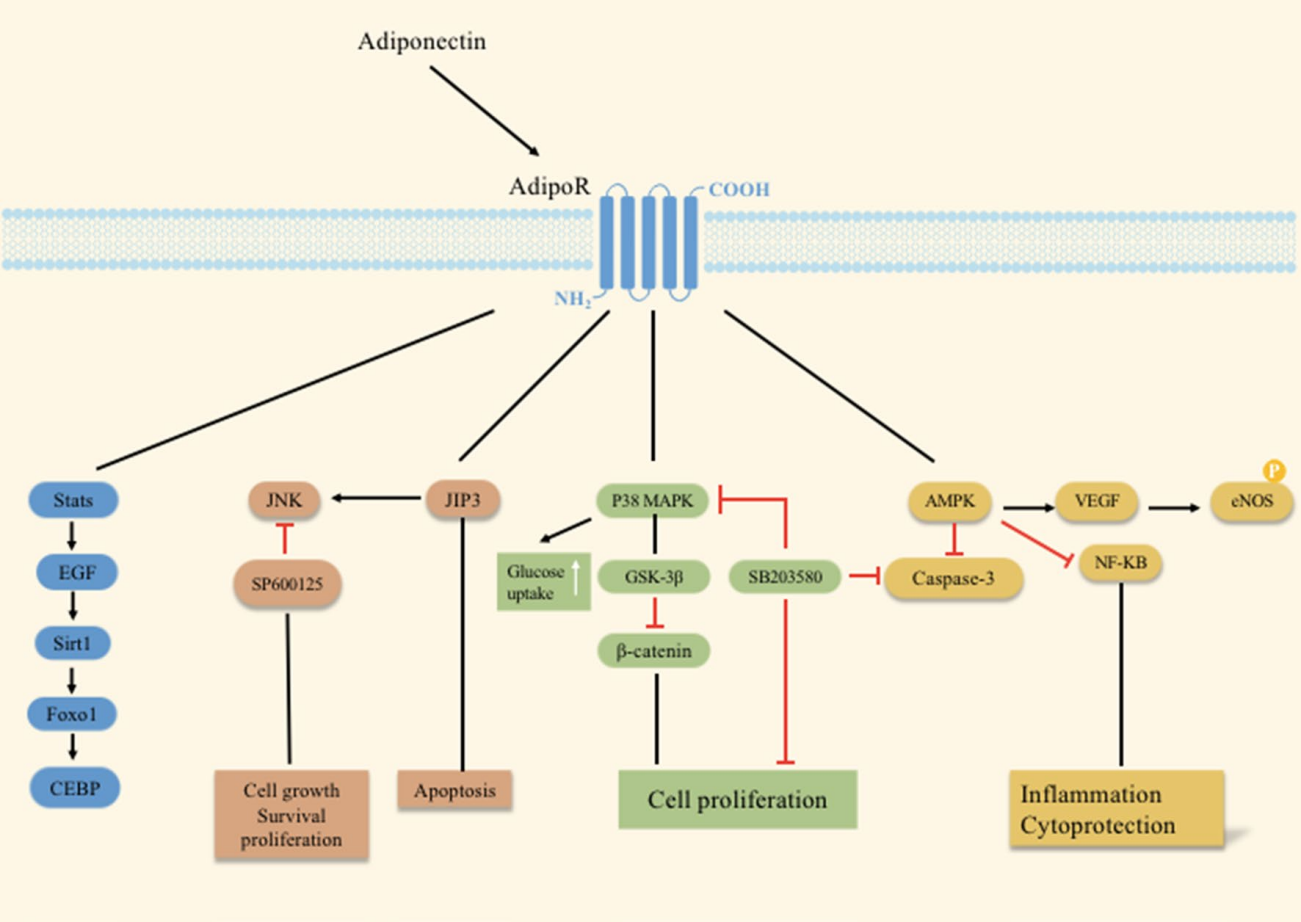
The role of adiponectin in various signaling pathways

Adiponectin is a hormone derived from adipocytes and is released into the circulation [13] in the form of full-length trimers, dimers (both of which are low molecular weight multimers), 18 or more high molecular weight multimers (HMW) [12, 92], and spherical moieties (gAD) [13]. It physiologically functions by activating downstream components of AMPK, P38-MAPK, c-Jun N-terminal kinase (JNK), and transcriptional regulatory nuclear factor-κB (NF-κB) signaling [14, 93–95]. However, the NAD + -dependent protein deacetylase SIRT1 and FoxO1-C enhancer binding protein α (EBPα) transcriptional complex affect the release of adiponectin [96, 97] (Fig. 2).

### The Regulation of Adiponectin in Energy Metabolism

It has been reported that changes of adiponectin in the central nervous system (CNS) may affect glucose metabolism in hippocampal neurons [98]; Interestingly, the occurrence of epilepsy involves the disorder of glucose metabolism. With the medical device of (18) F-fluorodeoxyglucose-positron emission tomography (18F-FDG PET) imaging to analyze the glucose metabolic changes in medial temporal lobe epilepsy patients with hippocampal sclerosis (mTLE‐HS), low signals could be routinely observed in the temporal and extratemporal areas [99]. Wang J et al. simultaneously used resting‐state functional Magnetic Resonance Imaging (rs fMRI) and 18F-FDG PET to study the coupling changes between brain metabolism and functional activity in mTLE-HS patients and found that hypometabolism, the fractional amplitude of low frequency fluctuations (fALFF) and increased regional Homogeneity (ReHo) areas are usually associated with the generation and transmission of epileptiform activity. There is a high coupling between resting-state spontaneous neural activity and glucose metabolism [100].

Since glucose cannot freely enter the cell through the lipid bilayer structure of the cell membrane, the transport function of glucose transporters (GLUT) on the cell membrane is necessary to achieve intracellular glucose intake. Glucose is an important energy source for the central nervous system. GLUT1 is expressed in CNS endothelial cells (ECs) and uncoupled with glycolysis in a Notch-dependent manner, and GLUT1 deficiency in stationary adult ECs leads to severe seizures with neuronal loss and CNS inflammation [101, 102]. GLUT1 deficiency syndrome (GLUT1 DS) caused by GLUT1 deficiency indicates the types of variable focal and multiple local seizure, and electroencephalography (EEG) manifestations [103]. The 2-[N-(7-Nitrobenz-2-oxa-1,3-diazol-4-yl) amino]-2-deoxy-D-glucose (2-NBDG) method and glucose uptake colorimetric method have shown that seizures could dramatically reduce neuronal glucose uptake and GLUT-3 expression [104]. However, study on hippocampal neurons from cultured primary rats and hippocampal slices from mice [98] have demonstrated that adiponectin enhances glucose uptake, glycolysis rate, and ATP production through the AMPK -dependent mechanism.

### The Regulatory Mechanisms of Adiponectin in Epilepsy

Apoptosis and inflammation induced by brain injury are pathogenic factors of epilepsy [105]; Nevertheless, adiponectin can protect cultured hippocampal neurons against KA-induced cytotoxicity, reduce reactive oxygen species, decrease apoptotic cell death, and inhibit KA-induced caspase-3 activation [106]. On the other hand, seizures also induce the production of inflammatory mediators, which trigger the activation of the NF-κB pathway to promote the progress of the disease in turn [107].

Adiponectin induces nuclear translocation of NF-κB p65 subunit and phosphorylation of MAPKs in dendritic cells [108]. Zhang et al. proposed that adiponectin triggers the proliferation of adult hippocampal neural stem/progenitor cells (hNSCs) through the p38MAPK/glycogen synthase kinase-3β (GSK-3β)/β-catenin cascade. P38MAPK inhibitor SB203580 not only eliminates this potentiation [109], but also reduces phosphorylated p38 (p-p38) in the PTZ-triggered rat epilepsy model [110], resulting in a decrease in caspase 3 level (Fig. 2). In addition, globular adiponectin plays anti-inflammatory and antioxidant roles in microglia through the AdipoR1/NF-κB signaling pathway [111].

P38 and JNK, another MAPK family member, are homologous protein-serine/threonine kinases, and selective targeting of JNK to P38 has been demonstrated as a potential therapeutic approach to epilepsy [112]. Tai and colleagues [113] found noticeable JNK overactivation in a TLE rat model induced by pilocarpine. The frequency of seizures obviously reduces with the use of a broad-spectrum nonspecific JNK inhibitor (SP600125), in contrast to the consequence for a nonspecific MAPK activator. Theselective inhibition of JNK-interacting protein 3 (JIP3) by lentivirus (LV-375JIP3-RNAi) attenuates the severity of seizure, consisting of reduced susceptibility of mice to the epileptogenic properties of KA, delayed first attack and decreased seizure duration. Apart from inhibiting JNK activation and neuronal apoptosis in the hippocampal CA3 region, underexpression of JIP3 has also been observed to delay the processes of PTZ-induced seizure firing [114]. Another concern is that neuronal damage after seizures is related to BBB leakage, and adiponectin maintains the integrity of the BBB and reduces the expression levels of vascular endothelial growth factor (VEGF), endothelial nitric oxide synthase (eNOS) and NF-κB in the hippocampus after KA-induced seizures in mice [115]. Notoriously, the BBB's integrity is critical in maintaining homeostasis and neuroprotection. We need more studies to confirm that reducing inflammatory stimulation by protecting the BBB may be a promising intervention or therapeutic strategy for epilepsy.

Antiepileptic drugs may be a potential factor affecting serum adipokine levels [116, 117]. Currently, VPA [118] is commonly used as a first-line antiepileptic drug in clinical practice, and its treatment time is negatively correlated with adiponectin level [119]. Clinical research has reported that adiponectin levels in children with idiopathic generalized epilepsy, obese children with idiopathic epilepsy, and adult epilepsy patients significantly decrease after valproate treatment [116, 119, 120]. Prospective evaluation of the long-term effects of the monotherapy of VPA and LTG on metabolic parameters in female epileptic patients for one year has found a remarkable decrease in adiponectin levels in the VPA group [121]. VPA-induced hypoadiponectinemia is significantly associated with weight gain and insulin resistance [119, 121]. The therapeutic concentration of VPA decreases adiponectin promoter activity in differentiated 3T3-L1 adipocytes, and inhibits gene expression of adiponectin in mature adipocytes [122].

Surprisingly, TPM, a novel antiepileptic drug, obviously decreases Leptin/Adiponectin (L/A) ratio and increases serum adiponectin level. Studies have shown that TPM increases energy metabolism and leads to weight loss in children with epilepsy [123]. The concentration of HMW adiponectin significantly increases in KD obese adolescents with no caloric restriction or a low-calorie diet for 6 months [124]. Treatment with antiepileptic therapy for 3 months for children with GLUT1 DS-resistant epilepsy aged 3–9 years [125], serum adiponectin level increases in the KD group (some of whom are also treated with other antiepileptic agents), compared with VPA monotherapy.

The specific adiponectin expression in different antiepileptic drugs may be bound up with multiple factors, including obesity, insulin resistance and molecular biology. With the in-depth study of clinical and basic experiments, we expect to deeply understand the specific mechanism of adiponectin to provide the theoretical basis for the clinically individualized treatment of epilepsy.

## Concluding Remarks and Future Perspectives

The adverse consequences of epilepsy affect people of all ages, and persistent seizures lead to accidents and even death. The control and treatment of epilepsy are essential to improve the patients' quality of life and to reduce mortality. Peripheral endocrine and metabolic factors regulate seizure threshold and seizure-related pathology by acting on neurons in the central nervous system, triggering intracellular signaling pathways or modulating neuronal activity. The dual role of leptin in epilepsy has attracted researchers' particular attention. On the one hand, the leptin receptor activates related signaling pathways to alleviate seizures and play a neuroprotective role. On the other hand, studies have reported the proconvulsant activity of Leptin in different models of epilepsy. The complex dual effects of leptin in treating epilepsy have brought about a more significant challenge, and its role in epilepsy control and treatment needs to be further studied. The association of leptin and adiponectin with epilepsy highlights the important role of these two adipokines in the pathophysiology of epilepsy pathogenesis. A large amount of evidence is helpful to better understand the complex biological mechanisms of leptin and adiponectin, and to provide ideas and a theoretical basis for the development and clinical application of effective hormone modulators in dealing with epilepsy in the future. We still need to further explore the role of adipokine imbalance in the adjusting effect of signal transduction in the pathogenesis of epilepsy, so as to find new methods and preventive measures for epilepsy.

## Data Availability

Not applicable.
